# Attention enhances stimulus representations in macaque visual cortex without affecting their signal-to-noise level

**DOI:** 10.1038/srep27666

**Published:** 2016-06-10

**Authors:** Mohammad Reza Daliri, Vladislav Kozyrev, Stefan Treue

**Affiliations:** 1 Neuroscience and Neuroengineering Research Laboratory, Biomedical Engineering Department, School of Electrical Engineering, Iran University of Science & Technology (IUST), Narmak, Tehran, Iran; 2 Cognitive Neuroscience Laboratory, German Primate Center, Goettingen, Germany; 3 School of Cognitive Sciences (SCS), Institute for Research in Fundamental Sciences (IPM), Niavaran, Tehran, Iran; 4 Visual Cognition Laboratory, University of Fribourg, Fribourg, Switzerland; 5 Faculty for Biology and Psychology, Goettingen University, Goettingen, Germany

## Abstract

The magnitude of the attentional modulation of neuronal responses in visual cortex varies with stimulus contrast. Whether the strength of these attentional influences is similarly dependent on other stimulus properties is unknown. Here we report the effect of spatial attention on responses in the medial-temporal area (MT) of macaque visual cortex to moving random dots pattern of various motion coherences, i.e. signal-to-noise ratios. Our data show that allocating spatial attention causes a gain change in MT neurons. The magnitude of this attentional modulation is independent of the attended stimulus’ motion coherence, creating a multiplicative scaling of the neuron’s coherence-response function. This is consistent with the characteristics of gain models of attentional modulation and suggests that attention strengthens the neuronal representation of behaviorally relevant visual stimuli relative to unattended stimuli, but without affecting their signal-to-noise ratios.

Functionally, visual attention enhances the representation of behaviorally relevant stimuli at the expense of other, irrelevant stimuli^1^. This enhancement is apparent in an increase in the responsiveness of neurons in visual cortex that prefer the aspects of the current attentional allocation in space and other dimensions, such as direction of motion^2^. Various mechanisms for this top-down modulation of sensory information processing have been proposed, including attentional modulation of synaptic weights^3^, changes in receptive field profiles^4^,^5^,^6^,^7^ and the selective enhancement of stimulus components by feature-based attention^8^,^9^, all mediated by influences from higher cortical areas, potentially through particular neurotransmitter systems^10^. Of particular relevance for this study is the suggestion that attention interacts with the system that converts the contrast of a visual stimulus into the sigmoidal, saturating contrast-response function (CRF) common to neurons in visual cortex. Such hypotheses are based on the observation that the CRF of neurons in extrastriate visual cortex are shifted horizontally by the allocation of spatial attention^11^,^12^ but note^13^ and that the perceptual effect of spatial attention is an enhanced perceived contrast of attended objects compared to physically identical but unattended stimuli^14^.

Such an effect of attention on the perceptual properties of a stimulus might be a special case restricted to contrast. Contrast might have this special role not only because it is encoded by a monotonic function (in contrast to circular stimulus properties such as orientation and motion direction) but also because it is the only stimulus property that influences responses in all neurons of visual cortex. The question of whether the attentional modulation of contrast responses is unique or is similar for all monotonically encoded stimulus properties is best addressed by investigating another monotonic stimulus-response relationship, namely the signal-to-noise ratio of stimuli. Similar to contrast a change in the signal-to-noise of a stimulus has widespread effects across sensory areas encoding the stimulus^15^,^16^.

To investigate these issues we used ‘motion coherence’, i.e. the proportion of dots undergoing uniform translation in a moving random dot pattern, as a measure of signal-to-noise ratio for visual motion stimuli. We recorded the responses of direction-selective neurons in area MT of macaque monkeys to such stimuli, presented in their receptive fields. Area MT is well known for its large proportion of direction-tuned neurons and is considered critical for visual motion perception. MT responses vary as a function of motion coherence. Specifically we wanted to know whether attention shifts the coherence-response function (akin to the effect seen for stimulus contrast) or whether it has a multiplicative effect on the coherence-response functions (akin to what has been observed for other responses, such as direction or orientation tuning). To address this question we compared the ability of three competing hypotheses of attentional modulation to fit our observations (response gain, activity gain, contrast gain; Fig. 1).

## Results

We determined the responses of 80 direction-selective neurons in area MT in two male, macaque monkeys as a function of the signal-to-noise (coherence) level of a moving random dot pattern inside their receptive fields when spatial attention was directed into or out of the receptive field. Figure 2 shows the time course of the activity for a typical example cell for two different coherence (signal-to-noise) levels (50 and 100%) for the preferred pattern in the two attentional conditions. The shaded area in each figure represents the attentional modulation for the sustained part of the responses used for the analysis (300–1070 ms after the stimulus onset). For this cell, we observed an attentional modulation of 32% and 34% for 50% and 100% motion coherence respectively.

The coherence-response function for each cell was created by taking the average responses during the sustained phase, for each level of coherence of the preferred pattern in each attentional condition and fitting the data sets with hyperbolic ratio functions. Figure 3A shows the coherence-response functions for four example cells. In the first two examples (first row), the R_max_ and R_bg_ are affected by attention while the C50 and s remain almost unchanged. The two other examples (second row) show cells with moderate and poor fitting, respectively.

Across our population the hyperbolic ratio function provided good fits with a median correlation of 95% (Fig. 3C) between the fitted functions and the data. Similarly, the median r.m.s. errors of the fitted curves were 10%, comparable to the values reported for contrast-response functions in area MT (9%)^17^.

We analyzed the population responses by determining the distribution of the indices of the fitting parameters (Fig. 3B). The distribution was significantly shifted for R_max_ (p = 3 * 10^−5^, paired t-test) and R_bg_ (p = 0.0019, paired t-test) while no significant shift was observed for the distribution of C50 (p = 0.48, paired t-test) and s (p = 0.51, paired t-test). On average R_max_ values were 19% higher and R_bg_ values were 15% higher when attention was directed outside the receptive field compared to the condition when attention was inside the receptive field, with no significant difference between the modulation of these two parameters (p = 0.55, paired t-test). The coherence-response functions based on the average parameters of the fits are shown in Fig. 4A. Figure 4C shows the normalized average population response as a function of coherence.

To further test the predictions of the coherence gain model vs. the response/activity gain model we used the data from trials with attention inside the receptive field to predict the responses for the *attending-outside* condition by either a multiplication (response/activity gain model) or a change in the C50 (coherence gain model) (for more details see^11^,^13^).

Figure 5 (top row) shows the correlation between the data and the best fits for each model. The correlations are high for all the models indicating that all three models provide a reasonable fit to the data. The dots fall along the unity line without a strong bias towards one of the models. To determine which model provides the best account of the data, we applied a partial correlation analysis^13^,^18^. The bottom panels in Fig. 5 show the partial correlation values for each model pairing. The shifts in medians show that both the activity and the response gain model provide a better fit to the data than the coherence gain model (p < 0.0001, ranksum test). Also, the data for the activity versus response gain model show that the median partial correlations (dashed lines) have shifted towards the activity gain model (p = 0.0073, ranksum test) documenting the superiority of this model. Thus, in agreement with our population analysis of the fit parameters, the activity gain model provides the best account for our data.

We determined the effect of attention on coherence-response functions in the influential Normalization Model of Attention^19^. The results of this analysis are plotted in Fig. 4B. In this model the normalization pool depends on the contrast of the stimulus and is independent of the stimulus’ orientations or directions. Thus, changing the coherency of the stimulus (i.e. its mixture of directions) will not affect the model’s behavior, creating a gain change in responses across coherences, in line with our experimental observations.

## Discussion

We investigated the effect of spatial attention on neuronal responses in macaque visual cortex to moving stimuli with different signal-to-noise ratios. Our data show a constant gain change to the responses of a given MT neuron, independent of the signal-to-noise ratio of the stimulus. While such multiplicative effect strengthens the responses to attended stimuli it leaves the overall shape of the response function unaffected. This is distinctly different from the horizontal shift a change in the contrast of a stimulus would cause. Thus our findings indicate that spatial attention creates an enhanced representation of attended stimuli without affecting their perceptual qualities.

The effect of spatial attention on neuronal tuning to a stimulus’ signal-to-noise ratio is akin to the multiplicative effects seen on other neuronal tuning curves, such as the tuning for orientation and direction of motion^20^,^21^. The singular exception from this gain-effect on tuning remains the shift in contrast-response functions observed in areas of both visual cortical pathways^11^,^12^. While not all studies support this shift-effect^13^ several aspects of stimulus contrast support its special status: under natural conditions contrast differs from stimulus property such as orientation and direction of motion in that contrast is not primarily a stimulus property, rather reflects the current lighting conditions. Therefore a change in contrast rarely reflects a change in stimulus and is interpreted perceptually as a change in the visibility or relative strength of an object without a change in its perceptual quality^14^. Furthermore contrast has the unique property of monotonically affecting neurons across all areas of visual cortex.

While the exact neuronal mechanisms underlying attentional gain changes and the shift in the contrast-response functions are unclear a number of recent models have focused on response normalization processes in cortical sensory processing^19^,^22^,^23^,^24^. Sensory normalization is a form of gain control in which neuronal responses are modulated in proportion to the activity of large pools of neighboring neurons. Depending on the relative size of the stimulus and the extent of the spatial attention focus (‘spotlight of attention’) the contrast-response function can be shifted or scaled upwards. For stimuli that vary their strength (i.e. contrast) the normalization signal will increase in parallel, thus limiting attentional effects at high contrasts. Correspondingly, for stimuli of constant contrast, where a second stimulus parameter is varied, the attentional modulation will be a constant gain effect, independent of the second stimulus parameter. Our observation of an activity gain effect on the signal-to-noise response function is consistent with these predictions of normalization models of attention.

Attention represents the core mechanism for selecting and enhancing a subset of the sensory input at the expense of the unattended input. As a consequence of such a mechanism the absolute representation of attended sensory inputs is enhanced or their strength relative to unattended inputs is increased. The Feature-Similarity Gain Model^2^,^25^ proposes that the underlying mechanism modulates the gain of sensory neurons depending on the similarity between the attended sensory aspects and the preference of a given neuron. If the neuron is activated by a single stimulus this strengthening is akin to a change in stimulus contrast. For noisy stimuli a similar effect on the signal level cannot be achieved as the gain change of the neuron will enhance both the signal and noise component of the stimulus. It is important to note that despite this inability the representation of an attended stimulus, even when it is noisy, is enhanced relative to unattended stimuli.

The difference in the attentional effects on the neuronal representation of stimulus contrast and of signal to noise levels outlined above suggests that perceptually changes in attentional allocation and stimulus contrast are difficult to discern, while this should be less ambiguous for changes in attentional allocation and signal-to-noise levels. For stimulus contrast such a perceptual confusion has been documented^14^. In a series of experiments subjects perceive the contrast of two spatially separated stimuli to be the same when the attended stimulus was of slightly lower contrast. A similar effect has been reported for motion coherence^26^. These perceptual effects are difficult to compare quantitatively with our physiological observations and it should be noted that Liu *et al*. did not include control conditions to rule out that the observed effect might reflect a change in perceived motion coherence, but instead is mediated by an increase in apparent stimulus contrast.

In summary, our data show that the magnitude of attentional response modulation in area MT does not vary with the coherence of moving random dot patterns, instead exerting a constant gain effect, independent of the signal-to-noise level of the stimulus. Unlike the attentional effects with varying contrast, attentional modulation does not seem equivalent to an increase in stimulus signal, but instead amplifies the activity of neurons to all levels of coherence, creating an enhanced representation of the overall stimulus while preserving its characteristic qualities.

## Methods

### Experimental Procedures

Coherence-response functions were recorded from 80 direction-selective MT neurons of two macaque monkeys. The cells were determined to be in MT by their anatomical position and their physiological characteristics (strength of directional-selectivity, receptive field size and position). Eye positions were measured using a video-based eye tracking system (ET-49, Thomas Recording). A custom-made program on an Apple Macintosh PowerPC controlled the stimulus presentation, eye positions and behavioral and neuronal data collections.

All animal procedures of this study have been approved by the responsible regional government office (Niedersaechsisches Landesamt fuer Verbraucherschutz und Lebensmittelsicherheit (LAVES)) and were carried out in accordance with all applicable laws and regulations. The animals were group-housed with other macaque monkeys in facilities of the German Primate Center in Goettingen, Germany in accordance with all applicable German and European regulations. The facility provides the animals with an enriched environment^27^ (incl. a multitude of toys and wooden structures), natural as well as artificial light, exceeding the size requirements of the European regulations, including access to outdoor space. All surgeries were performed aseptically under gas anesthesia using standard techniques, including appropriate peri-surgical analgesia and monitoring to minimize potential suffering. The German Primate Center has several staff veterinarians that regularly monitor and examine the animals and are consulted on procedures. During the study the animals had unrestricted access to food and fluid, except on days where data were collected or the animal was trained on the behavioral paradigm. On these days the animals were allowed unlimited access to fluid through their performance in the behavioral paradigm. Here the animals received fluid rewards for every correctly performed trial. Throughout the study the animals’ psychological and medical welfare was monitored by the veterinarians, the animal facility staff and the lab’s scientists, all specialized on working with non-human primates.

### Behavioral Task

The experimental paradigm was identical to the one developed by Martinez-Trujillo and Treue^11^. After manually mapping the receptive field of a neuron and determining the tuning curve and the preferred speed of the neuron, two moving random dot patterns (RDPs) were placed inside the receptive field and two additional RDPs in the opposite hemifield. One of the two stimuli (the target) moved toward the neuron’s anti-preferred direction at full coherence (anti-preferred pattern). The dots in the other stimulus (distracter) moved at the preferred direction of the neuron (preferred pattern). The motion coherence of the distracter differed from trial to trial. Two behavioral conditions were used: In each trial the monkey was cued to attend to the target stimulus, either an anti-preferred pattern inside the receptive field (*attending-inside* condition) or the same pattern outside the neuron’s receptive field (*attending-outside* condition). The monkeys’ task was to respond to a small direction or speed change of the target pattern by releasing the touch bar within a response time window (200–700 ms after the change) and ignore the changes of the other patterns (Fig. 6). The design ensures that the two attentional tasks are of equal difficulty because they are matched (the same eccentricity, same proximity to a neighboring stimulus, same task for the monkey in each condition etc.). Similarly, even though we are manipulating a stimulus variable that strongly affects the perceptual quality of the stimulus, this does not interact with the attentional load because the stimulus we are changing is behaviorally irrelevant while the target stimulus is always moving at full coherence.

For a successful completion of a trial the monkey’s gaze had to remain within 0.75 deg of the fixation point. The monkeys broke fixation in 35% of the trials. In 89% of the remaining trials, they performed correctly and in the remaining 11% of trials they responded outside the reaction time window. We found no significant differences in their performance between the two attentional conditions. Similarly there were no significant differences in the animals’ reaction times and hit rates for different levels of coherence (ANOVA, p = 0.31 for the hit rates and p = 0.98 for the reaction times). Note, that this is not surprising, since in both attentional conditions the attended stimulus was always moving at full coherence, with the unattended stimulus’ coherence varying between trials.

### Recording Techniques

Extracellular single unit action potentials were recorded using either single microelectrodes (FHC, Inc., Bowdoinham, ME) or a 5-channel microelectrode system (Mini Matrix, Thomas Recording, Giessen, Germany). The range of impedances was 0.5–2 *M*Ω. Transdural penetration was made using guide tubes. A data acquisition system (Plexon Inc., Dallas, TX) was used for recording and online sorting of the action potentials. Single units were isolated by window discrimination.

### Stimuli

Stimuli were random patterns of small white dots (RDPs) with a density of 10/deg^2^, moving within a stationary virtual circular aperture on a dark computer monitor (resolution: 40 pixel/deg) viewed from a distance of 57 cm. The refresh rate of the monitor was 76 Hz. At a given coherence level, the corresponding percentage of the dots moved coherently in one direction (here preferred direction of the neuron under study) and each of the remaining dots moved in a randomly chosen direction. All dots moved at the same speed. Note that this stimulus design is similar but not identical to the one developed by Movshon, Newsome and their colleagues^15^, in that our noise dots maintained the same speed as the signal dots. The dots in the anti-preferred pattern (the target) moved at full coherence in the anti-preferred direction.

### Data Analysis

For the data analysis we assessed neuronal *activity* during the sustained phase of the trials (300–1070 ms after the stimulus onset). Since response gain models of attention modulate only the response component of a neuron evoked by a sensory stimulus (leaving the spontaneous activity component unchanged), we also determined neuronal *responses* by subtracting the spontaneous activity of the neuron (recorded during trials where no stimulus was presented inside the neuron’s receptive field) from the measured activity of the neuron.

Just like the contrast-response function^11^,^12^,^13^,^16^, the neuronal responses or activities at different coherence levels can be approximated by a hyperbolic ratio function:


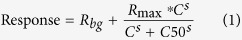


where R_bg_ is the neuron’s firing rate to the 0% motion coherence of the stimulus, R_max_ is the maximum firing rate of the neuron, C is the coherence level, C50 is the coherence where the firing rate is at the half-maximum and s is an exponent that determines the slope of the function. Each data set was fitted using a hyperbolic ratio function (Equation 1), with the ‘Nonlinear-Least-Squares’ method and the ‘Trust-Region’ algorithm, using the MATLAB Curve Fitting Toolbox. We weighted each data point according to the inverse of its standard error. The initial parameter values for R_bg_ and R_max_ were the minimum and the maximum data values respectively, the initial parameter of s was set to 1 and C50 was set to 50% as an initial value. The other initial parameters were left as the default values of MATLAB. To quantitatively analyze the effect of attention, attentional modulation indices (AMI) for each parameter of the fits were computed:


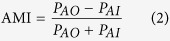


where P represents the relevant parameter in the hyperbolic ratio function, AO refers to the condition where attention is directed to the stimulus outside the receptive field, moving in the anti-preferred direction and AI refers to the condition where attention is directed inside the neuron’s receptive field.

**Figure 1:**
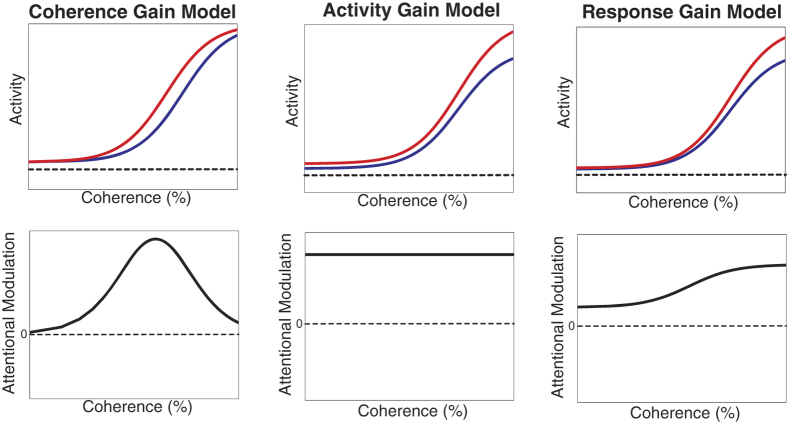
Three models for the effect of attention on coherence-response functions (Figure adapted from^4^). The top row shows the coherence-response functions in two attentional conditions in each model and the bottom row indicates the respective attentional modulation. Black horizontal lines in the top row indicate the spontaneous activity. Attention can shift coherence-response functions (coherence gain model), causing an attentional modulation that is particularly large for intermediate coherences (bottom left panel). Alternatively, attention can multiply the neuronal responses at all levels of motion coherence with a fixed factor (response gain model) or it can change neuronal activity by a fixed gain across all motion coherences (activity gain model).

**Figure 2:**
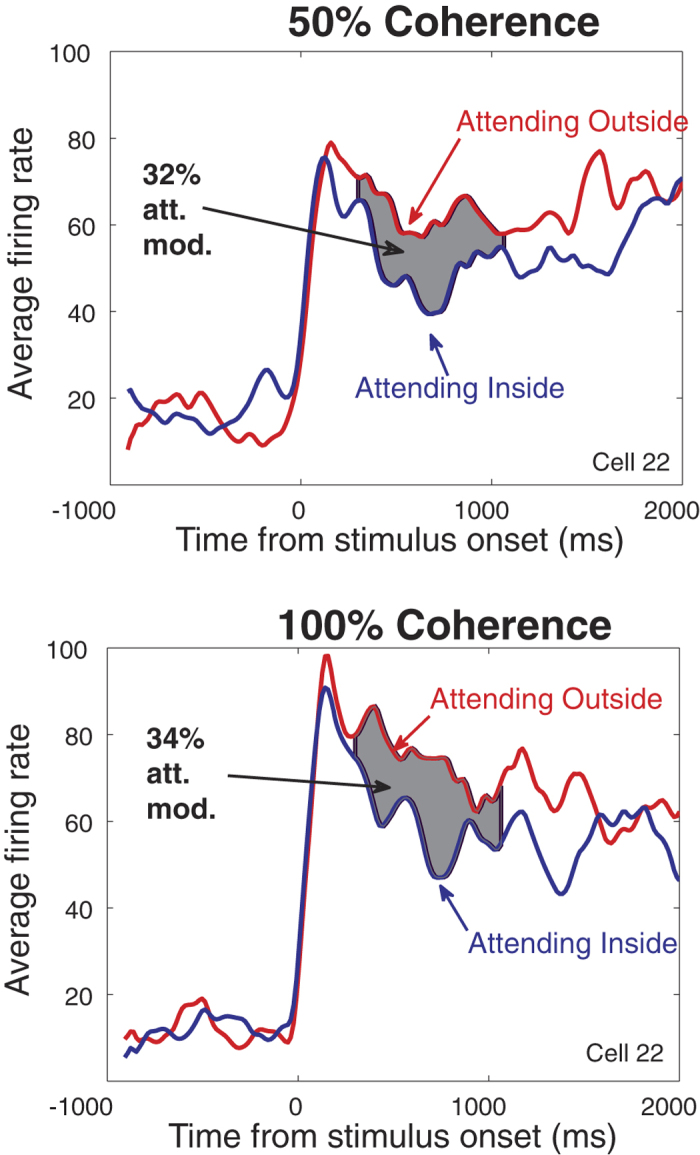
The time course of neuronal responses for an example cell: for two coherence levels of the preferred random dot pattern (top panel: 50%, bottom panel: 100%) in two attentional conditions (red: attending-outside; blue: attending-inside). Gray areas indicate the attentional modulation between the two conditions (32% and 34% for 50% and 100% motion coherence respectively) within the analyzed time window.

**Figure 3:**
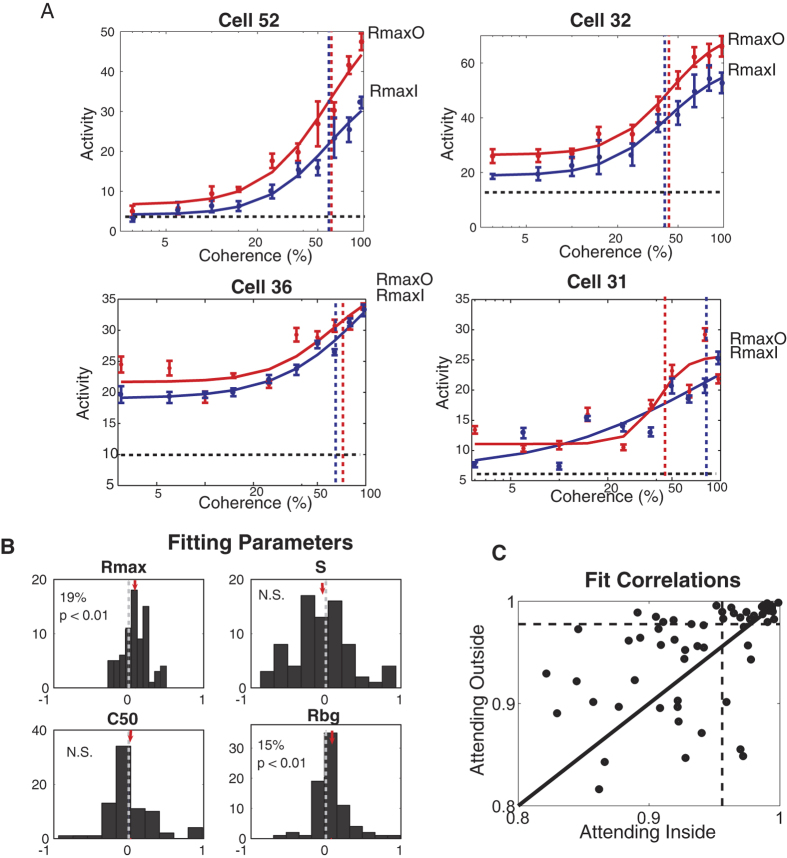
(**A)** Coherence-response functions for four example cells in two attentional conditions (red curves: attending-out condition; blue curves: attending-in condition). Dashed vertical lines mark the C50 location for each condition. (**B**) Attentional modulations for the fitting parameters of the coherence-response function (hyperbolic ratio function). Red arrows indicate the mean value of each histogram. Only the R_max_ (saturating response) and R_bg_ (response to 0% coherence) parameters are significantly affected by attention. (**C**) Correlation between the data and the fit (hyperbolic ratio function) for the attending-outside and attending-inside condition. Dashed lines indicate the median of correlation for each condition.

**Figure 4:**
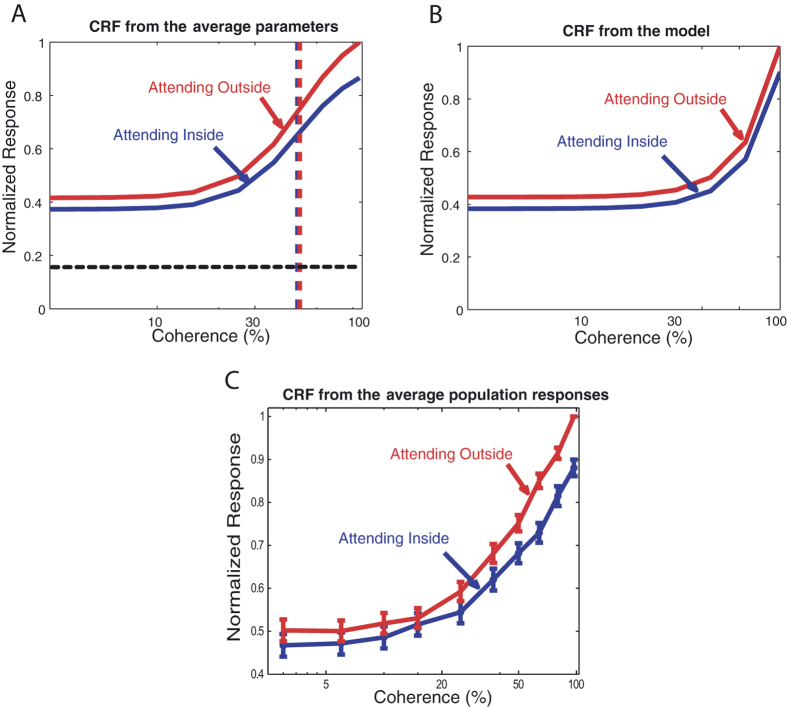
To determine the attentional modulation by changes in stimulus coherence in the Normalization Model of Attention^19^, we used code provided online by David Heeger. To create different levels of coherency, some percentage of the dots were manipulated to have the preferred feature (here the direction of motion) and the remaining dots were evenly distributed among other direction of motions. The result shows that the effect of attention on the coherence response functions is more likely to be multiplicative (activity gain model), rather than shifting the curve horizontally (coherence gain model). This matches the results we have obtained with the data we recorded from area MT. **(A**) Contrast response functions based on the average parameters of the fits for the two attentional conditions (blue: attending-inside; red: attending-outside). The red and blue vertical dashed lines mark the C50 for the two conditions and the horizontal black dashed line indicates the average spontaneous activity. The lack of a shift of the C50 clearly indicates that the data do not match the coherence gain model. (**B**) The coherence response functions for the two attentional conditions, obtained using the Normalization Model of Attention^19^. (**C**) The normalized average population response as a function of coherence. The blue curve indicates the attending-inside condition while the red one shows the attending-outside condition. The error bars are the standard errors of the mean across the population.

**Figure 5:**
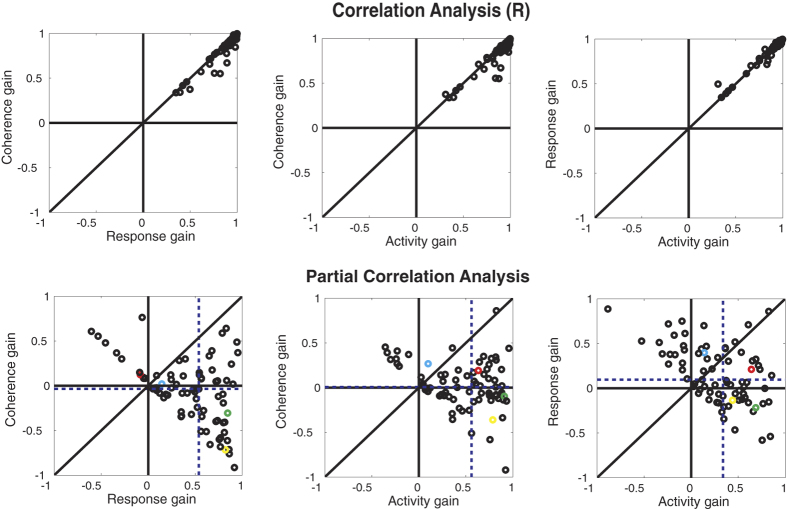
Correlation (top) and partial correlation (bottom) analysis for different models of attention on coherence-response functions. The correlation analysis shows that all of the models provide a good fit to the data (Note that the values plotted are correlations (R)). The partial correlation analysis indicates that an activity gain modulation is best able to account for the effect of attention on coherence-response functions, because the median of partial correlations (vertical dashed line) is shifted towards this model in the paired comparisons. The data points from the four example cells shown in Fig. 3A have been color-coded (Cell 52: Yellow, Cell 32: Green, Cell 36: Red, Cell 31: Cyan).

**Figure 6:**
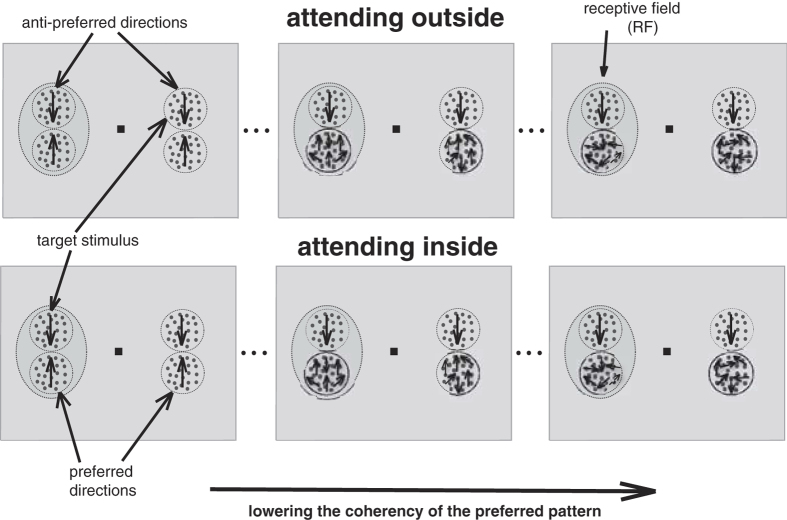
The experimental design for a cell preferring upward motion. In a given trial one pair of RDPs was placed inside a neuron’s RF (dashed ellipse) and one pair outside the RF. One of each pair (in- and outside RF) moved in the preferred direction (preferred pattern) of the neuron and the other in the anti-preferred direction (anti-preferred pattern). An anti-preferred pattern was the target in both attentional conditions (inside and outside). From left to right, the coherence of the preferred pattern was systematically decreased to determine the coherence-response function in the two attentional conditions.

The data are compared against three models of attentional modulation: the response gain model, activity gain model and coherence gain model (Fig. 1). The effect of attention in the coherence gain model is a horizontal shift of the coherence-response functions. In the response gain model, attention increases a neuron’s gain effectively multiplying its response (i.e. the neuron’s activity after subtracting the baseline response) to all motion coherence levels by the same factor. The activity gain model similarly changes the gain but the multiplicative modulation affects the overall activity of the neuron, not just the response to the stimulus.

As the three fitted functions have very similar shapes, we determined their differences by looking at the response variance that is explained by each fit. The partial correlation method eliminates the effect of correlation between the different functions used for the fits. The partial correlations for the Activity Gain, the Coherence Gain and the Response Gain predictions have the following general form:


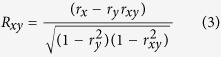


where r_x_ is the correlation of the data with the x model prediction, r_y_ is the correlation of the data with the y model prediction and r_xy_ is the correlation between the two prediction models. The superscript of x and y can be one of the three models used to fit the data.

To test the influential Normalization Model of Attention^19^, we used code provided online by David Heeger (http://www.cns.nyu.edu/heegerlab/content/software/attentionModel/attentionModel.zip). To create different levels of coherency, some percentage of the dots had the preferred feature (here the direction of motion) and the remaining dots were evenly distributed among other direction of motions.

## Additional Information

**How to cite this article**: Daliri, M. R. *et al*. Attention enhances stimulus representations in macaque visual cortex without affecting their signal-to-noise level. *Sci. Rep.* **6**, 27666; doi: 10.1038/srep27666 (2016).
